# Hybrid coffee cultivars may enhance agroecosystem resilience to climate change

**DOI:** 10.1093/aobpla/plab010

**Published:** 2021-02-25

**Authors:** Emily Pappo, Chris Wilson, S Luke Flory

**Affiliations:** 1 School of Natural Resources and Environment, University of Florida, 103 Back Hall, Gainesville, FL 32603, USA; 2 Agronomy Department, University of Florida, 1676 McCarty Hall B, PO Box 110500, Gainesville, FL 32611, USA

**Keywords:** Climate change, coffee, *Coffea arabica*, cultivars, water stress

## Abstract

Anthropogenic climate change is predicted to cause shifts in temperature and precipitation patterns that will be detrimental for global agriculture. Developing comprehensive strategies for building climate resilient agroecosystems is critical for maintaining future crop production. Arabica coffee (*Coffea arabica*) is highly sensitive to the quantity and timing of precipitation, so alterations in precipitation patterns that are predicted under climate change are likely to be a major challenge for maintaining coffee agroecosystems. We assessed cultivar selection as a potential component of more resilient coffee agroecosystems by evaluating water stress responses among five Arabica coffee cultivars (clonal hybrids H10 and H1 and seedling lines Catuai 44, Catuai, and Villa Sarchi) using a precipitation reduction experiment in the highlands of Tarrazú, Costa Rica. During the first harvest (eighteen months after planting), plants under the rainout treatment had 211 % greater total fruit weight and over 50 % greater biomass than under the control treatment, potentially due to protection from unusually high rainfall during this period of our experiment. At the second harvest (30 months after planting), after a year of more typical rainfall, plants under rainout still produced 66 % more fruit by weight than under control. The magnitude of the responses varied among cultivars where, at the first harvest, H10 and H1 had approximately 92 % and 81 % greater fruit production and 18 % and 22 % greater biomass, respectively, and at the second harvest H10 had 60 % more fruit production than the overall average. Thus, our findings suggest that the hybrid lines H10 and H1 are more resilient than the other cultivars to the stress of high soil moisture. Overall, our results indicate that stress due to higher than average rainfall could impair coffee plant growth and production, and that cultivar selection is likely to be an important tool for maintaining the viability of coffee production, and the resilience of global agroecosystems more generally, under climate change.

## Teaser

Could your daily cup of coffee be threatened by climate change? Results from an experiment in Costa Rica found that coffee yields can, in fact, be impacted by changing rainfall patterns, but those effects can be mitigated by planting more resilient cultivars. The study experimentally reduced precipitation for five coffee cultivars and measured yield over two harvests, ultimately finding that the F1 hybrid cultivars in the experiment outperformed the other cultivars under both reduced and ambient conditions. These findings suggest the selection of more resilient cultivars can help maintain agroecosystem production in the face of a changing climate.

## Introduction

Many agroecosystems are facing complex challenges associated with maintaining economic sustainability under climate change (Calzadilla *et al.* 2013). In addition to driving punctuated high temperature stress, climate change is causing significant fluctuations in annual precipitation patterns, including extreme events that result in drought and flooding stress for crops (Calzadilla *et al.* 2013; Rosenzweig *et al*. 2014; Parker *et al*. 2020). Climate change is also contributing to enhanced pest, disease and weed pressure (Yan *et al*. 2017). Proposed solutions to these challenges often fall on crop breeders, with research expanding towards identification of the physiological traits underlying stress tolerance and development of methods aimed at producing more resilient crop genotypes (Kole 2013), and thus more resilient agroecosystems. As part of these efforts, it is important to evaluate traditional and emerging cultivars under prospective environmental conditions to inform breeding programs, improve farm-level cultivar selection and reveal new strategies to help meet the demand for global crop production under a changing climate.

Increasing extremes of temperature and precipitation associated with climate change present a significant threat to agroecosystems, especially for crop species with highly restrictive environmental requirements (Parry *et al*. 2004). In particular, Arabica coffee is a globally significant commodity (Pendergrast 1999) that is expected to be impacted by climate change because it is reliant on particular temperature regimes for the production of quality beans (Camargo 1985; dos Santos *et al*. 2015) and is sensitive to the timing and quantity of rainfall (Haarer 1958; Alègre 1959; Maestri *et al*. 1977). Coffee sustains the livelihoods of an estimated 100 million people (Pendergrast 1999) on 12.5 million farms (Browning 2018) in over 60 countries (ICO 2018). In 2018, global Arabica production exceeded 13 billion pounds (5.9 billion kg; ICO 2018), and coffee consumption is increasing in both importing and exporting countries (ICO 2018). Despite the potential for effects of climate change on production (Bunn *et al*. 2015), and the global economic significance of coffee, we have a poor understanding of how climate change will affect coffee plant performance and yield. As such, coffee provides a good case study to explore strategies and solutions for climate change mitigation in agroecosystems.

Modelled climate change shifts in temperature and precipitation patterns (Bunn *et al*. 2015; Ovalle-Rivera *et al*. 2015) predict a decrease in suitable land area for coffee production of up to 50 % (Bunn *et al*. 2015), with variable effects on different coffee-producing regions (Ovalle-Rivera *et al*. 2015). An annual average increase of 2 °C is projected across coffee-producing regions, in combination with variable changes in seasonal rainfall (Ovalle-Rivera *et al*. 2015). For example, some regions of East Africa and the Andes are projected to experience overall increases in precipitation, while parts of Mesoamerica and Brazil are expected to have less precipitation (Ovalle-Rivera *et al*. 2015). In some areas, these predictions can be even more complex. For example, Costa Rica is expected to have an overall reduction in annual precipitation but is likely to experience more frequent extreme rainfall periods, with rainfall at times exceeding 50 mm/day (Imbach *et al*. 2018).

Changes in environmental conditions may be detrimental for coffee production because coffee plants require specific temperatures and amounts of rainfall during developmental and reproductive life stages to produce high quality yields (Haarer 1958; Maestri *et al*. 1977; dos Santos *et al*. 2015). Like many tropical plants, the geographical distribution of coffee is limited by low temperatures associated with high elevation or cooler climates (DaMatta *et al*. 2006). However, high temperatures at low elevation in many tropical areas hasten fruit development such that there may be negative effects on bean quality (Camargo 1985; dos Santos *et al*. 2015). As a result, most high-quality coffee is grown at moderate to high elevations in the tropics. In addition, the precipitation requirements of coffee are highly specific, with coffee requiring over 1200 mm of precipitation per year and consistent annual wet/dry cycles (Alègre 1959). Specifically, a 2- to 4-month dry period is required for flowering (Haarer 1958; Maestri *et al*. 1977), and there must be adequate soil moisture during fruit development (Carr 2001).

The threat of climate change to coffee production has spurred the development of multiple farm-level strategies, such as moving production to new areas that could gain suitability (Bunn *et al*. 2015; Ovalle-Rivera *et al*. 2015), adding value through third-party certifications or a shift to ‘specialty’ coffee, which provides a price premium for high quality (Vellema *et al*. 2015), or adding shade trees to reduce the stress from variable environmental conditions (Toledo and Moguel 2012; Cerda *et al*. 2017; Padovan *et al*. 2018; Rahn *et al*. 2018). However, improved selection of cultivars for planting may be the most effective farm-level strategy to improve agroecosystem resilience if cultivars can be identified that maintain both production and quality under future climate conditions.

Coffee cultivars are selected for planting based primarily on yield, quality, pest and disease tolerance, cost and historical and cultural considerations. However, there is a distinct lack of information on how coffee cultivars vary in their responses to current and future environmental conditions. For example, although both drought and excess rainfall (i.e. waterlogging, saturation) may stress coffee, most currently available information is based on how cultivars perform under more typical climate conditions (World Coffee Research, 2019). Some research has explored underlying mechanisms of how coffee generally responds to water stress without focussing on differences among cultivars (Meinzer *et al*. 1990; Ramos and Carvalho 1997; DaMatta 2004; Silveira *et al*. 2014).

To maintain coffee production under climate change, selecting coffee cultivars that can maintain yields under different levels of precipitation would be useful in building agroecosystem resilience by broadening the range of conditions under which the agroecosystem can function. The goal of our experiment was to better understand variation in coffee cultivar responses to changes in precipitation by using rainout shelters that experimentally manipulated precipitation in the field over two harvest cycles. We sought to answer the questions: 1) To what degree will experimentally reducing rainfall impact coffee plant performance? and 2) Do responses of coffee plants to experimentally reduced rainfall vary among cultivars? We hypothesized that while overall coffee plant performance would be impacted by the experimentally manipulated rainfall conditions, there would be variability among the cultivars in their responses. Better understanding of variation in cultivar responses to altered precipitation will support the use of cultivar selection as a tool to build resilience in coffee agroecosystems.

## Methods

### Study region

**Figure 1.**
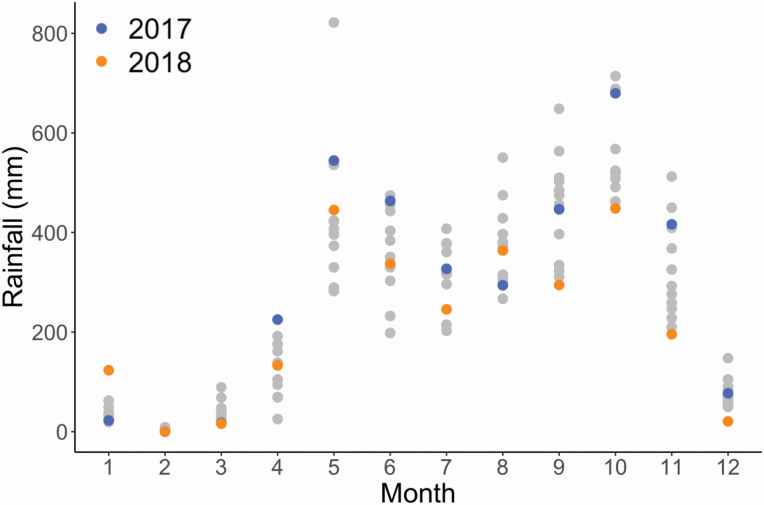
Total monthly rainfall in the study region from 2006 to 2018. The years of the experiment are highlighted in blue (2017) and orange (2018), and the rest are light grey (2006–16). Rainfall data from the nearest Climate Engine (Huntington *et al.* 2017) collection site is shown.

Our experiment was conducted on an active coffee farm at approximately 1750 m elevation near near Santa María de Dota in the coffee growing region of Tarrazú, Costa Rica. Santa María de Dota has an average annual temperature of 19 °C and its climate is characterized by distinct rainy and dry seasons, with the rainy season typically extending from April/May through October/November (Icafe 2019). In the first year of our experiment (2017), the site received an estimated 3514 mm of rainfall with several months where rainfall levels were particularly high relative to the past decade. For example, in the months leading up to planting (May and June 2017), the site received 31 % and 30 % more rain than average, respectively, than historical averages and in October 2017, the site received 18 % higher rainfall in part due to heavy rains brought on by Hurricane Nate. During the second year of our experiment, rainfall patterns were closer to the regional average of 2923 mm, experiencing an estimated 2625 mm (Fig. 1).

### Study site

We established our experiment on a 30 m × 25 m former pasture that was occupied by locally common native and non-native grass species. Soils at the site had an average pH of 4.72, and soils in this volcanic highland region are primarily classified as Andosols (FAO and UNESCO 1988). We selected the site due to its uniform slope (~ 29 %), aspect (northeast/east-northeast), and sun exposure (no trees in the immediate research area). In addition, the site is surrounded by coffee under production, indicating the site’s suitability for the experiment.

### Experimental design

**Figure 2.**
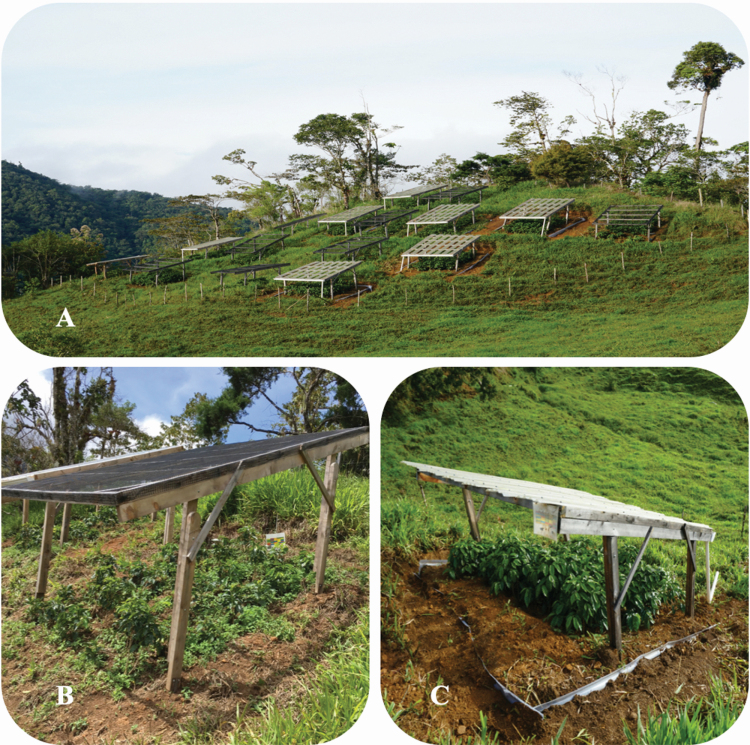
(A) The research site near Santa Maria de Dota. (B) Control shelter with mesh roofing and (C) rainout shelter with polycarbonate roofing, gutter, pipes and in-ground plastic barrier.

To determine how the performance and production of multiple coffee cultivars were affected by variation in rainfall, we implemented a common garden experiment with coffee transplants using a randomized block and split plot design with ambient (control) and reduced rainfall (via rainout shelters) treatments and five coffee cultivars. In total, there were eight blocks, each with a control and a rainout plot, for a total of 16 plots (Fig. 2A). Rainout shelters (Fig. 2C) were erected with wood frames and polycarbonate roofing that provided 75 % aerial coverage. Rainout shelters also had gutters and PVC pipes that directed the rainfall away from the plots and 1 m in-ground plastic barriers buried on three sides of each rainout plot (not installed on the downhill side) to 0.75 m deep with 0.25 m remaining aboveground to reduce subsurface and surface flow of water, respectively. The rainout shelters greatly reduced rainfall but not by the 75 % suggested by the roof cover because rain was driven into the plots from the sides by wind and the belowground and surface flow barriers reduced, but did not entirely exclude, subsurface and surface flow of water. To compensate for the shade produced by the rainout shelters and other potential unintended shelter effects, control plots were covered by identical wooden structures but with mesh netting instead of polycarbonate roofing (Fig. 2B).

Each plot was randomly planted with seven individuals of each of five cultivars: H10 (also known as Milenio), H1 (also known as Centroamericano), Catuai 44, Catuai and Villa Sarchi. These cultivars were chosen based on production potential (H10, H1, Catuai 44) and common usage in the area (Catuai, Villa Sarchi). H10 and H1 are F1 hybrid cultivars (Rume Sudan × Catimor/Sarchimor) developed by French Agricultural Research Centre for International Development (CIRAD) that were reportedly high quality and high yield, with resistance to some pests and diseases (WCR 2019). However, the F1 hybrids cost approximately twice as much as cultivars of more common seedlings. Catuai (Cat) is a commercially common Mundo Novo × Caturra cross developed by the Instituto Agronômico (IAC) in Brazil that is described as having good quality and average yields and is susceptible to common pests and diseases (WCR 2019). Catuai 44 (Cat44) is a line of Red Catuai developed by IAC that is believed to be more drought tolerant. Villa Sarchi (VS) is a Bourbon mutation bred by the Instituto del Café de Costa Rica (Icafe) with similar quality, yield and susceptibility to Catuai (WCR 2019).

The plants were all 1 year old and were planted on 20 July 2017. Catuai, Catuai 44 and Villa Sarchi were sourced from a nursery in San Marcos, Costa Rica where they were grown from seed, and H10 and H1 were sourced from a commercial facility in Orosí, Costa Rica where they were grown as vegetatively propagated clones. Thirty-five individuals total per plot were planted on a grid at an even spacing of 0.5 m × 0.5 m, which is closer than the industry standard (Winston *et al*. 2005; Bittenbender and Smith 2008; Wintgens 2009) but allowed for more data to be collected from each plot while the plants were small. After the first coffee harvest, we removed a subset of the plants so we could collect data on plant biomass from the harvested plants and could increase plant spacing to the typical production standard of 1 m × 1.5 m. We reduced the number of plants per plot to three individuals of four cultivars (12 individuals total per plot) for long-term evaluation. Due to space constraints in the plots, and because we had originally included both Catuai and Catuai 44 in the experiment, which are functionally similar, we removed Catuai entirely.

### Environmental conditions

To evaluate the effects of the rainout shelters on soil moisture, 10 soil moisture measurements (percent volumetric water content [%VWC]; HydroSense II, 20 cm probe, Campbell Scientific, UT, USA) were taken in each plot every 2 weeks using the standard calibration for the HydroSense system. To determine if the rainout and control shelters similarly reduced light availability, light measurements (photosynthetically active radiation [PAR]; Apogee 10, Apogee Instruments Inc., UT, USA) were taken every 3 months. Concurrent measurements were taken inside each structure above the plants and outside the structures in full sun to calculate percent light reduction in the plots. To evaluate the potential effects of the shelters on temperature (°C) and humidity (%RH), we used hourly temperature and humidity data collected from data loggers (HOBO U23 Pro v2 Temperature/Relative Humidity Data Loggers, Onset Computer Corp., MA, USA) installed in each shelter during June 2019 through January 2020. The data loggers were housed in upside down plastic pots (approximately 22 cm wide × 20 cm deep) that had vents cut in the sides and were hung in the centre of plots from wires midway between the underside of the shelter frames and the plant canopy, which protected the loggers from direct sun and approximated the conditions experienced by the plants.

### Data collection and analysis

In December 2018, 18 months after planting, all coffee fruit was harvested from each tree, counted and weighed. In addition, a subset of the plants from each plot (three of each cultivar in each plot, 240 plants total) were removed at ground-level, dried in ovens (60 °C) to constant mass, and weighed. For the second harvest in January 2020, 30 months after planting, all coffee fruit was again harvested from each tree and weighed.

Environmental variables, including soil moisture, light, temperature and humidity, were analysed using linear models (lm) fit with ordinary least squares using the ‘lm’ function in R (Version 3.5.2). For light availability, temperature and humidity, we used a model that included both a term for treatment as well as a second order Fourier Series expansion to account for the periodic (diurnal) patterns in the data (Wilson *et al*. 2017). Fruit and biomass data were analysed using the lmer function in the lme4 package (Version 1.1–19). Specifically, we fit varying-intercept/varying-slope linear mixed effect models (e.g. Gelman and Hill 2007; Zuur *et al*. 2011) where we included a fixed effect indicator of rainout treatment (where 0 = control, and 1 = rainout), and then let both intercepts and slopes (treatment effects) vary by cultivar identity (*n* = 5, i.e. we treated cultivar as a random effect). These models generated partially pooled estimates of each cultivar’s performance under both treatment conditions, allowing us to assess among-cultivar heterogeneity and obviating the need for separate post-hoc adjustments for multiple comparisons (Gelman *et al*. 2012).

For inference, we report both the estimate and standard error of the fixed effect of rainout treatment, noting where the resulting t-score exceeded two (usually associated with a *P* < 0.05 threshold), but focussing more on the magnitude of the effect and its uncertainty and variation with cultivar identity. Additionally, we compared the variance components associated with the cultivar random effects on the SD scale against the magnitude of the treatment effect to quantitatively infer the extent of heterogeneity among cultivars.

## Results

**Figure 3.**
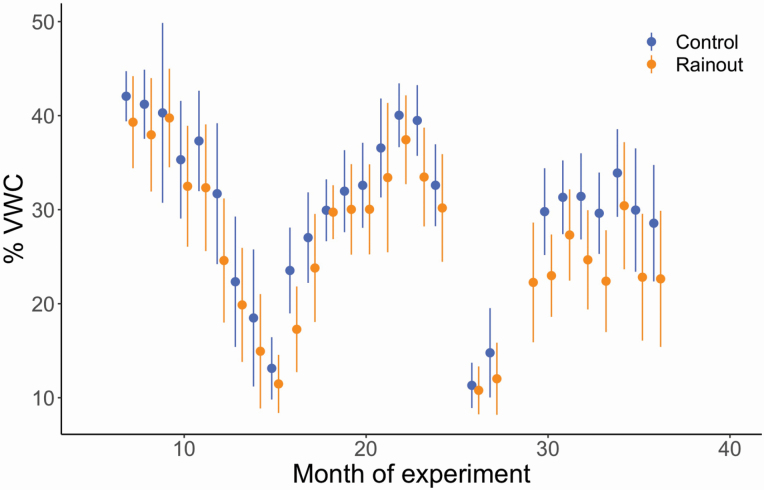
Mean monthly soil moisture (% volumetric water content, VWC) in control and rainout treatments. %VWC of the rainout plots was on average 14 % less than the control plots over the course of the experiment (*P* < 0.0001). Data points jittered for clarity.

Volumetric soil moisture of the rainout treated plots was on average 14 % less (SE: 0.17 %) than the control plots over the course of the experiment (*P* < 0.0001), with the average rainout plot having 24.8 % VWC and the average control 28.7 % VWC, although the magnitude of the difference varied seasonally (Fig. 3). The reduction in light availability (PAR) for rainout (26.4 %) and control shelters (26.3 %) was very similar (+0.1 SE: 0.48 %, *P* = 0.846). Temperature slightly differed with treatment, with rainout plots having a somewhat higher average temperature of 20.2 °C than the control’s average of 19.5 °C (+0.7 °C SE: 0.02 °C, *P* < 0.0001, **see**  **Supporting Information—Fig. S1**). There were similarly small but statistically significant differences in relative humidity between the treatments. Rainout plots had an average RH of 83.9 % while control plots averaged 86.5 % (-2.6 % SE: 0.07 %, *P* < 0.0001).

**Figure 4.**
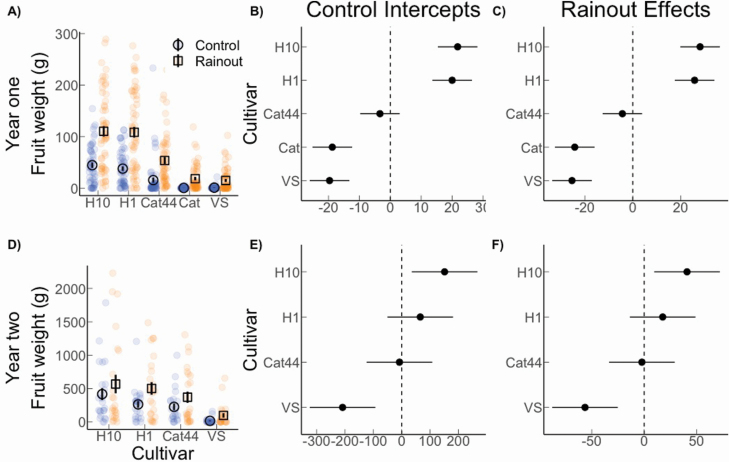
Fruit weight 18 months (A) and 30 months (D) after planting was positively affected by the rainout treatment across the cultivars. The coefficient plots for control intercepts (B, E) and rainout effects (C, F) show the estimates as offsets from the overall mean (for more detail, **see**  **Supporting Information Tables S1 and S2**). Where the 95 % confidence interval does not overlap 0, we can infer that the estimate differed significantly from the mean.

Total fruit weight and fruit count per tree were highly correlated (R^2^: 0.937) so we only report fruit weight here. The results of the first harvest showed that rainout treatment had a significant effect on total fruit weight across all cultivars (Fig. 4A) where average fruit weight under rainout was 211 % greater than under the control treatment. Average fruit weight per plant was 62.2 g under rainout (SE: 14.7 g) and 20.0 g (SE: 11.4 g) under the control treatment, with a fixed effect estimate of 42.2 g (SE: 14.7; Fig. 4A). In addition, there were clear differences among the cultivars in responses to the treatment. For instance, Cat and VS produced essentially no fruit under control conditions but produced approximately as much under rainout as the next biggest producer, Cat44, produced under control. H10 and H1 had approximately twice as much fruit weight production per plant than Cat44 regardless of treatment (Fig. 4A). When the estimates for each cultivar are compared with the overall mean, a clear pattern emerges where Cat and VS perform significantly below baseline and H10 and H1 perform significantly above baseline in both rainout and control treatments (Fig. 4B and C). The estimated variance component of the varying treatment effects by cultivar (i.e. varying responses to rainout) was 24.8 g (SD scale), roughly 50 % of the average treatment effect (fixed effect reported above).

The results of the second harvest showed a less pronounced difference in fruit weight between plants under control and rainout treatments. Overall, average production for plants under rainout conditions was 384.7 g, which is 66.3 % greater than the control average of 231.3 g (Fig. 4D) with a fixed effect estimate of 153.4 (SE: 121.0 g). However, though the treatment effect is in the same direction as the first harvest, there was larger uncertainty, which is consistent with the rainfall being more normalized in the second year (Fig. 1). There also was variation in response among the cultivars, though again less pronounced than during the first harvest, with an estimated variance component of the varying treatment effects by cultivar of 42.8 g (SD scale), roughly 30 % of the fixed effect of treatment. Specifically, H10 produced higher than average yields and VS produced significantly below average yields under both control and rainout conditions, while H1 and Cat44 production did not differ significantly from the baseline (Fig. 4E; Fig. 4F).

**Figure 5.**
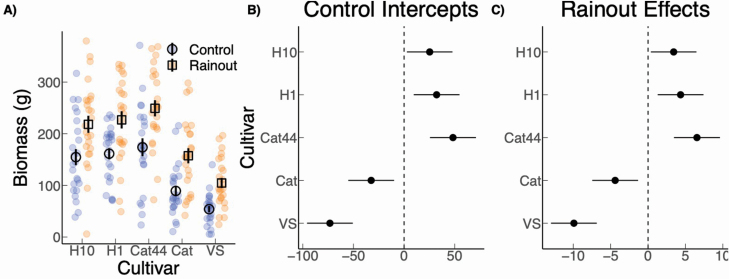
Aboveground plant biomass 18 months after planting for the five original coffee cultivars in the experiment (A). Biomass was positively affected by the rainout treatment across the cultivars, though to a lesser degree than fruit weight (Fig. 4A). Coefficient plots for control intercepts (B) and rainout effects (C) show estimates as offsets from the overall mean (for more detail, **see**  **Supporting Information Table S3**). Where the 95 % confidence interval does not overlap 0, we can infer that the estimate differed significantly from the mean.

The rainout treatment also had an overall positive effect on biomass production. While the effect size was lower than for fruit count and weight, uncertainty was much smaller, with a fixed effect estimate of 64.8 g (SE: 21.6 g; Fig. 5A). Average aboveground biomass production under rainout was 191.4 g (SE: 27.6 g), which was 51 % higher than the average of 126.6 g (SE: 21.6 g) under the control treatment. Overall, it appears that genetic potential for biomass differs among cultivars regardless of treatment (Fig. 5B and C). The estimated variance component for varying treatment responses by cultivar was only 7.0 g (SD scale), which is minor compared to the fixed effect (average) of treatment (64.8 g). In addition, Cat44 showed the greatest biomass accumulation, followed by H1 then H10.

## Discussion

Global agroecosystems are predicted to be significantly affected by fluctuations in environmental conditions, including temperature and rainfall, under climate change. For many crops, inconsistent rainfall is the primary driver of decreases in plant performance and productivity. Here, we found that experimentally reducing precipitation improved coffee plant fruit production and growth, particularly over their first year in the field when rainfall was unusually high. In general, fruit production was greater under the rainout treatment, and there was substantial variation among cultivars, where the F1 hybrid cultivars H10 and H1 showed the greatest overall yields during year one and H10 also had the greatest yields at the second harvest. While the rainout treatment had a positive effect on overall biomass production at the end of the first year, there was minimal biomass variation among cultivars in response to the rainout treatment, suggesting that the variability in biomass accumulation overall was likely due to genetic variability in potential for biomass among cultivars. Overall, our results suggest that environmental stress due to unusually high rainfall can impair coffee plant performance but that cultivar selection, particularly the F1 hybrids tested here that can maintain production under wet conditions, may be a critical tool for maintaining coffee production under climate change. In addition, our results challenge the idea that producers would necessarily need to accept a tradeoff between productivity and resilience by suggesting that there are opportunities for high productivity under suboptimal conditions.

All of the cultivars performed better under rainout conditions than control, a result that was particularly pronounced at the first harvest. Higher than average rainfall during the first several months of the experiment likely created saturated soil conditions and, although it was not logistically possible to collect plant stress physiology data, the rainout treatment appeared to protect the plants from water stress during that time. During a time of high rainfall, higher than average soil moisture can lead to hypoxic or anoxic conditions where plants are no longer able to maintain necessary oxygen-requiring metabolism (Silveira *et al*. 2014). When oxygen levels are low, plants must activate the anaerobic fermentive pathway, which only provides small amounts of energy, and reactive oxygen species that can damage or even kill the plant may be formed (Silveira *et al*. 2014). Rainfall was closer to average during the second year of the experiment and performance of control treated plants was more similar to plants under the rainout treatment.

Environmental variables other than soil moisture, such as light, temperature and humidity, can affect coffee plant performance (Wintgens 2009), but we have little evidence that they differed between the control and rainout treatments and influenced results of the experiment. Average light availability was similar between the treatments but it is possible that the polycarbonate and mesh roofing provided different light quality or temporal variation in light. Mean temperatures under rainout and control treatments were below thresholds for irreversible damage to photosynthesis (Martins *et al*. 2016; Rodrigues *et al*. 2016) but under both treatments there were periods of above-threshold temperatures. Temperatures in rainout plots exceeded the threshold of 42 °C on 3 % of the days where temperature data was logged, while control temperatures never reached that high. However, these observations would suggest that rates of photosynthesis would have been more heavily suppressed in rainout plots and would not explain the greater production under these conditions. Mean humidity under rainout and control were both above the optimum threshold (Wintgens 2009), and there was slightly higher humidity under control conditions, possibly due to higher soil moisture. Ultimately, we think it is clear that soil moisture was the driving environmental factor underlying differences in plant performance across the treatments.

Studies investigating the effects of high soil moisture on coffee plant performance have been rare. Silveira *et al*. (2014) showed that Red Catuai and Mundo Novo seedlings varied in response to waterlogging, with Mundo Novo seedlings showing a slightly greater tolerance to waterlogged soils. Cultivars may also vary in their responses to other environmental conditions. For example, there is evidence for significant inter-cultivar variation in response to higher temperatures (Teixeira *et al*. 2015), which could be useful to consider in tandem with findings regarding water stress tolerance because projections for Central America suggest an average increase of 2 °C in annual mean temperature by 2050 (Imbach *et al*. 2018). Our findings add to the understanding of coffee cultivar responses to water stress and help inform planting decisions under climate change.

Though the F1 hybrid cultivars H10 and H1 were more productive under variable rainfall, there are some key challenges to their adoption at the farm level, primarily the cost of implementation. Due to the required clonal propagation of F1 hybrids, they are labour and resource intensive to produce and thus are often priced about twice as high as more commonly available seedlings that are grown from seed. Profit margins are often slim on coffee farms (Beuchelt and Zeller 2011; Bravo-Monroy *et al*. 2016; Clay *et al*. 2018) so the price difference may be difficult for producers to manage. However, despite challenges to implementation, the F1 hybrid cultivars show resistance to nematodes, coffee berry disease and coffee leaf rust (CLR; WCR 2019). CLR in particular is a major challenge for coffee production (Avelino *et al*. 2015) and is predicted to worsen under climate change (Ghini *et al*. 2011; Avelino *et al*. 2015; Bebber *et al*. 2016). They also typically perform well in regards to quality as well as yield (WCR 2019), two components that may lead to higher farm income (Bravo-Monroy *et al*. 2016; Clay *et al*. 2018). Despite higher upfront costs, it is possible that the combination of high production, resistance to common pests and diseases, and tolerance to variability in precipitation could make these cultivars, and others that share these characteristics, promising options for improving resilience in some coffee agroecosystems.

Our experiment provides important information about coffee cultivar responses to variable precipitation but there are outstanding questions about climate change effects on coffee. First, it is important to better understand how climate change may impact coffee quality, which affects the value of a coffee harvest. Both high temperatures (Camargo 1985; dos Santos *et al*. 2015) and high moisture during post-harvest processing (Barbosa *et al*. 2012; dos Santos *et al*. 2015) may lower coffee quality. However, little is known about how soil moisture variability impacts quality and whether such effects differ by cultivar. Second, our experiment examined the response of plants to different precipitation levels during two harvest seasons but it is unknown whether hybrid precocity may have contributed to our findings so longer-term studies with more highly controlled environmental conditions are needed. Finally, a critical research need is to explore whether the cultivars that responded best to waterlogging stress would also respond similarly to water deficit stress. Cultivars that maintain productivity under the broadest range of conditions are likely to be a key component of climate resilient agroecosystems.

## Conclusions

Our results demonstrate that experimentally reducing precipitation during periods of high rainfall enhanced Arabica coffee performance. Fruit production varied by cultivar, with the F1 hybrid cultivar H10 having the greatest performance over the duration of the study. Variation among cultivars in response to water stress indicates that greater rainfall under climate change may threaten coffee production and that cultivar selection, particularly the selection of cultivars that can maintain production under variable rainfall conditions, may be a useful tool for mitigating those impacts. More broadly, our results suggest that selecting cultivars for farm-level environmental conditions and future climatic conditions could enhance development of climate resilient agroecosystems.

## Supporting information

The following supporting information is available in the online version of this article—

**Figure S1.** Minimum, mean and maximum temperatures (°C) for control (blue) and rainout (orange) plots, binned by month. The panels on the left show daytime temperatures (between 06:00 and 17:00) and those on the right show night-time temperatures (between 17:01 and 05:59). Data points jittered for clarity.

**Table S1.** Estimates of year one fruit weight (g) for each cultivar under control and rainout as offsets from the overall mean, as represented graphically in Fig. 4B and C.

**Table S2.** Estimates of year two fruit weight (g) for each cultivar under control and rainout as offsets from the overall mean, as represented graphically in Fig. 4E and F.

**Table S3.** Estimates of biomass (g) for each cultivar under control and rainout as offsets from the overall mean, as represented graphically in Fig. 5B and C.

**Table S4.** ANOVA table for the model (using the packages lmer and lmerTest) showing the fruit weight results by treatment (‘trt’) and cultivar (‘cult’) for fruit weight over both years of the experiment. These model results indicate that the effects of treatment and cultivar were each significant (*P* < 0.05) and there was not a significant interaction between treatments.

**Table S5.** ANOVA table for the model (using the packages lmer and lmerTest) showing the biomass results by treatment (‘trt’) and cultivar (‘cult’). These model results show that the effects of treatment and cultivar were each significant (*P* < 0.05) and there was not a significant interaction.

plab010_suppl_Supplementary_Material

## Data Availability

Data is publicly available via DataDryad at https://doi.org/10.5061/dryad.3tx95x6fq

## Notes

**Agroecology & Environment. Chief Editor:** Amélie Gaudin
