# Supplementary material for: Hybrid coffee cultivars may enhance agroecosystem resilience to climate change
Source: AoB Plants. 2021 Feb 25;13(2):plab010. doi: 10.1093/aobpla/plab010 (PMC7991896; doi:10.1093/aobpla/plab010)
Supplement: plab010_suppl_Supplementary_Material [file plab010_suppl_supplementary_material.pdf]

Supporting Information (SI) for:
Hybrid coffee cultivars may enhance agroecosystem resilience to climate change

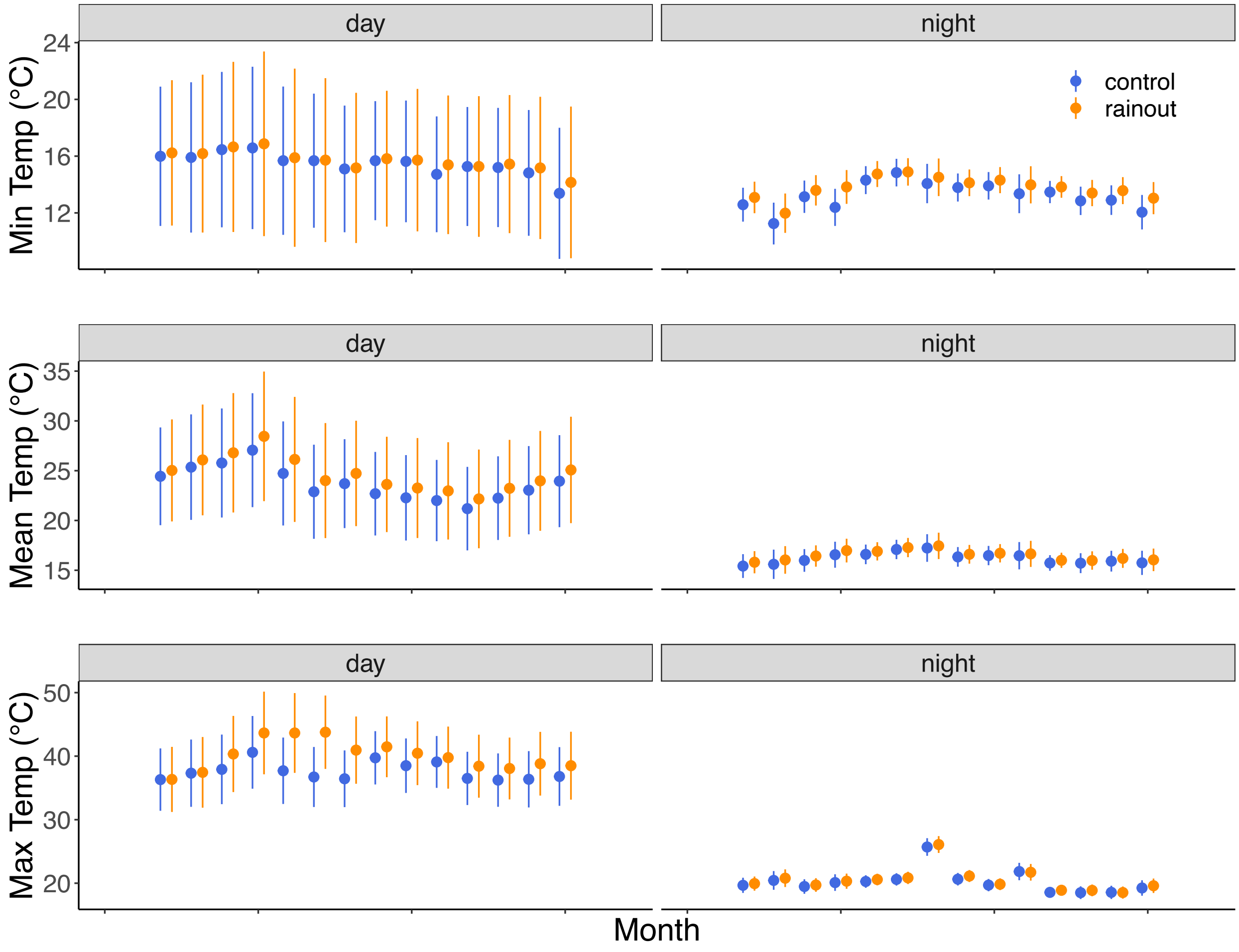

Supporting Information Figure S1 - Minimum, mean, and maximum temperatures (°C) for control (blue) and rainout (orange) plots, binned by month. The panels on the left show daytime temperatures (between 06:00 and 17:00) and those on the right show nighttime temperatures (between 17:01 and 05:59). Data points jittered for clarity.

Cultivar	Control	Rainout
H10	21.782910	28.108135
H1	20.012182	25.823231
Cat44	-3.357078	-4.331892
Cat	-18.778147	-24.230862
VS	-19.659867	-25.368612

Supporting Information Table S1 – Estimates of year one fruit weight (g) for each cultivar under control and rainout as offsets from the overall mean, as represented graphically in Figs. 4B and 4C.

Cultivar	Control	Rainout
H10	151.163378	40.896774
H1	65.364242	17.684089
Cat44	-7.965862	-2.155139
VS	-208.561757	-56.425724

Supporting Information Table S2 – Estimates of year two fruit weight (g) for each cultivar under control and rainout as offsets from the overall mean, as represented graphically in Figs. 4E and 4F.

Cultivar	Control	Rainout
H10	25.29650	3.424621
H1	32.14134	4.351270
Cat44	48.28624	6.536954
Cat	-32.49331	-4.398919
VS	-73.23077	-9.913926

Supporting Information Table S3 – Estimates of biomass (g) for each cultivar under control and rainout as offsets from the overall mean, as represented graphically in Figs. 5B and 5C

	Sum Sq	Mean Sq	NumDF	DenDF	F value	Pr(>F)
**trt**	208136.9	208136.89	1	14.1077	5.1816	0.0389
**cult**	3044649.4	761162.36	4	723.0876	18.9494	0.0000
**trt:cult**	235540.3	58885.08	4	723.0005	1.4660	0.2108

Supporting Information Table S4 – ANOVA table for the model (using the packages lmer and lmerTest) showing the fruit weight results by treatment (“trt”) and cultivar (“cult”) for fruit weight over both years of the experiment. These model results indicate that the effects of treatment and cultivar were each significant ($p < 0.05$) and there was not a significant interaction between treatments.

	Sum Sq	Mean Sq	NumDF	DenDF	F value	Pr(>F)
**trt**	28593.131	28593.1309	1	14.0032	9.1857	0.0090
**cult**	592163.574	148040.8934	4	216.0277	47.5591	0.0000
**trt:cult**	3916.759	979.1898	4	216.0277	0.3146	0.8681

Supporting Information Table S5 – ANOVA table for the model (using the packages lmer and lmerTest) showing the biomass results by treatment ("trt") and cultivar ("cult"). These model results show that the effects of treatment and cultivar were each significant ($p < 0.05$) and there was not a significant interaction.
